# Potential Role of CXCL13/CXCR5 Signaling in Immune Checkpoint Inhibitor Treatment in Cancer

**DOI:** 10.3390/cancers14020294

**Published:** 2022-01-07

**Authors:** Ching-Hung Hsieh, Cheng-Zhe Jian, Liang-In Lin, Guan-Sian Low, Ping-Yun Ou, Chiun Hsu, Da-Liang Ou

**Affiliations:** 1 Department of Clinical Laboratory Sciences and Medical Biotechnology, College of Medicine, National Taiwan University, Taipei City 10048, Taiwan; r08424005@ntu.edu.tw (C.-H.H.); lilin@ntu.edu.tw (L.-I.L.); 2 Graduate Institute of Oncology, College of Medicine, National Taiwan University, Taipei City 10051, Taiwan; d10453002@ntu.edu.tw (C.-Z.J.); chsu1967@ntu.edu.tw (C.H.); 3 Department of Animal Science and Technology, National Taiwan University, Taipei City 10672, Taiwan; b07606044@ntu.edu.tw; 4 Institute of Biomedical Informatics, National Yang Ming Chiao Tung University, Taipei City 11221, Taiwan; s93042.md09@nycu.edu.tw; 5 Department of Medical Oncology, National Taiwan University Cancer Center, Taipei City 10672, Taiwan; 6 YongLin Institute of Health, National Taiwan University, Taipei City 10672, Taiwan

**Keywords:** immune checkpoint inhibitor, CXCL13/CXCR5, tertiary lymphoid structure

## Abstract

**Simple Summary:**

Immunotherapy is currently the backbone of new drug treatments for many cancer patients. CXC chemokine ligand 13 (CXCL13) is an important factor involved in recruiting immune cells that express CXC chemokine receptor type 5 (CXCR5) in the tumor microenvironment and serves as a key molecular determinant of tertiary lymphoid structure (TLS) formation. An increasing number of studies have identified the influence of CXCL13 on prognosis in patients with cancer, regardless of the use of immunotherapy treatment. However, no comprehensive reviews of the role of CXCL13 in cancer immunotherapy have been published to date. This review aims to provide an overview of the CXCL13/CXCR5 signaling axis to summarize its mechanisms of action in cancer cells and lymphocytes, in addition to effects on immunity and cancer pathobiology, and its potential as a biomarker for the response to cancer immunotherapy.

**Abstract:**

Immune checkpoint inhibitors (ICIs), including antibodies that target programmed cell death protein 1 (PD-1), programmed death-ligand 1 (PD-L1), or cytotoxic T lymphocyte antigen 4 (CTLA4), represent some of the most important breakthroughs in new drug development for oncology therapy from the past decade. CXC chemokine ligand 13 (CXCL13) exclusively binds CXC chemokine receptor type 5 (CXCR5), which plays a critical role in immune cell recruitment and activation and the regulation of the adaptive immune response. CXCL13 is a key molecular determinant of the formation of tertiary lymphoid structures (TLSs), which are organized aggregates of T, B, and dendritic cells that participate in the adaptive antitumor immune response. CXCL13 may also serve as a prognostic and predictive factor, and the role played by CXCL13 in some ICI-responsive tumor types has gained intense interest. This review discusses how CXCL13/CXCR5 signaling modulates cancer and immune cells to promote lymphocyte infiltration, activation by tumor antigens, and differentiation to increase the antitumor immune response. We also summarize recent preclinical and clinical evidence regarding the ICI-therapeutic implications of targeting the CXCL13/CXCR5 axis and discuss the potential role of this signaling pathway in cancer immunotherapy.

## 1. Introduction

Immunotherapy is one of the most successful forms of treatment for cancer patients [1]. Understanding and identifying tumor-specific cytotoxic T cells and tumor-associated antigens have provided additional insight into the effective design of immunotherapies capable of killing cancer cells [2,3,4,5,6]. In addition to the generation of tumor-specific effector cells that can attack cancer cells, the attraction of immune cells, especially effector cells, to the tumor microenvironment represents a critical step in cancer therapy. To effectively attack established cancer cells, effector immune cells must be able to traffic to the tumor site. Chemokines were originally identified by their chemoattractant abilities to attract white blood cells [7,8,9,10]. During inflammation or an immune response, various leukocytes recognize specific chemokines, which attract them to specific sites where they mediate effects. Consequently, chemokines maintain immune system homeostasis, regulate the innate or adaptive immunity response, and recruit leukocytes to the specific site [11,12]. Additionally, chemokines can activate and prime lymphocytes, triggering their transformation into effector cells that attack tumor cells and suppress tumor proliferation [13,14]. Chemokines are also capable of suppressing immune cell function, limiting their abilities to attack tumor cells [15,16,17].

Chemokines are small peptides with molecular weights of 8–10 kDa that can specifically circulate, retain, and activate some immunocompetent cells [18,19]. Chemokines are chemotactic cytokines that mediate activity through interactions with specific G protein-coupled receptors (GPCRs) with seven transmembrane domains, known as chemokine receptors [20]. The activation of the chemokine/chemoreceptor signaling pathway induces cell proliferation, migration, homing, survival, and gene expression [21,22,23,24]. The interaction between chemokines and their receptors activates a complex and plastic signaling pathway, which is crucial for the regulation of specific leukocyte subpopulation responses. Variations in chemokine expression levels can result in a wide range of functions with consequential effects on the treatment response to immune checkpoint inhibitors (ICIs) among patients with cancer. Elevated or reduced expression levels of different chemokines or chemokine receptors have been associated with diverse responses to immunotherapy across multiple cancer types [25,26,27]. Chemokine activation and the downstream function of signaling effectors influence almost all of the processes involved in the immunotherapy treatment response in cancer.

C-X-C motif chemokine ligand 13 (CXCL13), also known as B lymphocyte chemoattractant, was initially detected in stromal cells of B cell follicles, associated with the recruitment of B cells and T cells subsets [28,29]. CXCL13 has also been identified as a critical homeostatic chemokine that is expressed in associated lymphoid tissues, and B cells and follicular T helper (T_FH_) cells form B and T cell connective zones according to CXCL13 gradients.

C-X-C motif chemokine receptor type 5 (CXCR5) is the main and only receptor for CXCL13 and mediates the functions of chemokines through specific downstream interactions [30,31,32]. The interaction between CXCR5 and CXCL13 can induce the entry of T or B cells into lymphoid organs and integrin expression, and CXCR5-deficient mice present with serious immune system defects [32,33]. An increasing number of studies have identified that various influences of CXCL13 expression levels prognosis in patients with cancer regardless of immunotherapy treatments [34,35,36,37,38].

The CXCL13/CXCR5 axis is linked with tumor development, progression, proliferation, and invasion. Abnormally active CXCL13/CXCR5 signaling enhances cancer cell growth through complex and different mechanisms in breast cancer [39,40], intestinal cancer [32,41], lung cancer [42], prostate cancer [43,44,45], oral squamous cell carcinoma [46,47], renal cell carcinoma [48], neuroblastoma [49,50], and lymphoma [51,52]. In this review, therefore, we discuss the CXCL13/CXCR5 axis and summarize its mechanism of action in cancer cells and lymphocytes, its contributions to immunity and cancer pathobiology, and its role in response to immunotherapy and potential to serve as a biomarker.

## 2. The CXCL13/CXCR5 Signaling Axis

**Figure 1:**
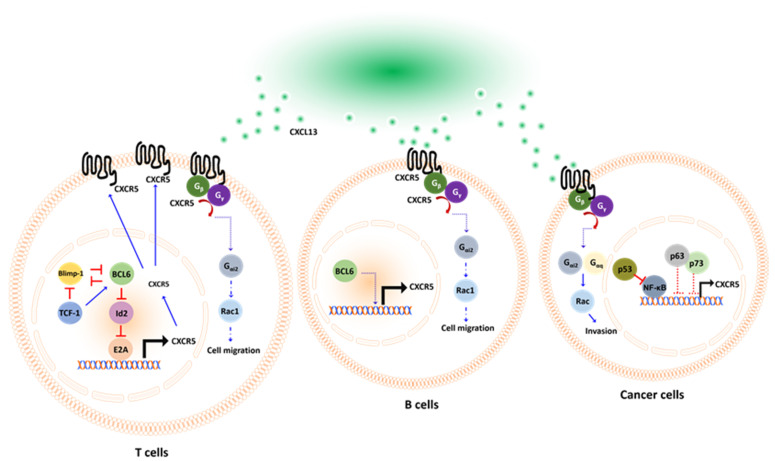
Schematic representation of CXCL13/CXCR5 axis in T cells, B cells, and cancer cells, respectively. CXCL13 acts as a ligand that specifically binds to its receptor CXCR5. Upon activation, the GDP bound G_α_ protein subunit is replaced with a GTP molecule. This leads to the dissociation of G_αq_ or G_αi2_ from the G_β_–G_γ_ dimer, mediating downstream signal activation. CXCL13 promotes chemotaxis in T cells and B cells. However, in cancer cells, CXCL13 promotes migration and invasion. The transcriptional factors involved in CXCR5 expression are diverse in T or B cells and cancer cells. In T cells, BCL6 regulates the CXCR5 expression via a repressor of repressor circuit. While in cancer cells, p53 regulates CXCR5 indirectly by suppressing NF-κB.

The specific downstream signaling pathway activated by the binding of CXCR5 with CXCL13 remains unclear and is likely dependent on the GPCR activity of CXCR5 [53]. El-Haibi et al. reported that G_α13_ co-immunoprecipitated with CXCR5 under CXCL13-treated conditions in prostate cancer cells [54]. However, G_αq_ and G_αi2_ co-immunoprecipitate with CXCR5 in the absence of CXCL13 but dissociated in the presence of CXCL13. When a GPCR binds with a ligand, the GDP bound to the small G protein is replaced with a GTP, activating the small G protein and resulting in dissociation from the GPCR. The dissociated small G protein mediates further cellular signal transduction [55]. G_αq_ and G_αi2_ but not G_α13_ dissociate from the GPCR CXCR5 upon binding with CXCL13, suggesting that G_αq_ and G_αi2_ were the major active small G proteins involved in CXCL13/CXCR5 signaling activation. Additional evidence was provided about small interfering RNA (siRNA) targeting G_αq_, G_αi2,_ and G_α13_ [54]. CXCL13-dependent cell invasion in prostate cancer (PCa) cells was significantly inhibited by the application of siRNA targeting G_αq_ and G_αi2_, indicating that the CXCL13/CXCR5/G_αq_ signaling pathway regulates cellular chemotaxis. Small G protein downstream kinases Rac and RhoA were determined upon cancer cells receiving CXCL13. They found CXCL13 activated Rac but not RhoA, and this was in G_αq_ and G_αi2_ dependent manner. Small G protein downstream kinases Rac and RhoA were determined upon cancer cells receiving CXCL13. They found CXCL13 activated Rac but not RhoA, and this was in G_αq_- and G_αi2_-dependent manner. The precise mechanism of CXCL13/CXCR5 signaling is still unclear in B cells and T cells. There is only rare evidence suggesting that small G proteins would play an important role in CXCL13/CXCR5 signaling. In B cells, Han et al. demonstrated that G_αi2_ is the dominant factor determining B cell mobility [56]. The G_αi2_^−/−^ B cells respond poorly to CXCL13 treatment and fail to migrate toward chemokine sites in a filter plate assay. When G_αi2_^−/−^ B cells were transplanted into recipient mice, they failed to migrate to the lymph node. In T cells, Hwang et al. demonstrated that G_αi2_^−/−^ cells were characterized by poor mobility and impaired trafficking capabilities [57]. Cell migration declined, on average, approximately 4- to 7-fold in G_αi2_^−/−^ CD4^+^ and CD8^+^ T cells compared with wild-type cells when exposed to chemotaxis conditions. They assessed the morphology of secondary lymphoid structures in G_αi2_^−/−^ mice by immunohistochemical staining and identified the disrupted expression of T cells in the marginal zones, the failure of germinal center formation, and a smaller lymphoid structure size in G_αi2_^−/−^ mice compared with wild-type mice. The detailed mechanism of how CXCL13/CXCR5 signaling works is represented in Figure 1 below. In summary, the CXCL13/CXCR5 signaling in T cells, B cells, and cancer cells are similar. In T cells and B cells, CXCL13/CXCR5 stimulates their chemotaxis, and this is in G_αi2_ dependent manner. The downstream signaling of G_αi2_ in CXCL13-treated T cells and B cells is still not fully clear, probably are Rac1, Rac2, or other Rho GTPase [58]. In cancer cells, CXCL13/CXCR5 regulates cell invasion and migration, and those are in G_αq_ and G_αi2_ /Rac dependent manner.

Transcriptional factor p52/RelB in the NF-κB pathway had been reported as a transcriptional factor of CXCL13 in macrophage [59] and B cells [60,61] in mice lacking p52 or with a RelB defect in B-cell follicles and germinal centers formation [62]. NF-κB proteins are also key transcriptional factors of immune checkpoint PD-L1 [63,64,65], suggesting that NF-κB would simultaneously control immune checkpoint blockade response and T/B cell recruitment. However, different transcriptional machinery controls the expression of CXCR5. In T cells, BCL6 activates CXCR5 expression by repressing repressor pathways [66]. Transcriptional factor E2A is directly bound to enhancer regions of CXCR5 to activate its expression [67,68]. However, Id2/Id3 dampened CXCR5 expression by antagonizing E2A [68,69]. BCL6 acts as a transcriptional repressor that directly inhibits Id2 expression in T cells, which, in turn, activates E2A activity and then stimulates CXCR5 expression [66,70]. Interestingly, BCL6 and Blimp-1 are mutually exclusive and antagonize each other [71]. If Blimp-1 dominates BCL6 in this mutual competition, naïve CD4^+^ T cells will differentiate into Th1 cells that do not express CXCR5 [71], in contrast, if upstream transcriptional factor T cell factor 1 (TCF-1) is triggered, naïve CD4^+^ T cells would differentiate into T_FH_ and express CXCR5 [72]. This differentiation occurs because TCF-1 is a transcriptional factor that simultaneously suppresses Blimp-1 [73,74] and activates BCL6 [75]. In CD8^+^ T cells, transcriptional regulators TCF-1, BCL6, E2A, Blimp-1, and Id2/Id3 also work collaboratively to shape CXCR5 expression with the same mechanism, as described in T_FH_ cells [68]. In cancer cells, Mitkin et al. identified that p53 indirectly suppressed CXCR5 expression in the MCF breast cancer cell line. Silencing of p53 with shRNA significantly restored CXCR5 expression in breast cancer cells. This was because p53 suppressed transcriptional factor NF-κB, which directly transactivates CXCR5 by interacting with the promoter region [76]. The expression of CXCR5 is regulated by the transcription factors BCL6 and Blimp-1. BCL6 drives the expression of CXCR5, whereas Blimp1 plays an inhibitory role. BCL6 and Blimp1 antagonize each other to regulate CXCR5 expression in T cells. [71] (Figure 1).

In addition to CXCL13/CXCR5, G_ai2_ is also involved in CXCR3 downstream signaling upon receiving its chemotaxis ligands CXCL9, CXCL10, and CXCL11. The CXCR3 has two variants, CXCR3A and CXCR3B. CXCL4, CXCL9, CXCL10, and CXCL 11 activate G_ai_ in CXCR3A but not CXCR3B [77]. Of those G_ai_ subunits, G_ai2_ is the most dispensable for T cells’ CXCR5 response to CXCL9, CXCL10, and CXCL11 [78].

The regulator of G protein signaling (RGS) acted as a GTPase-activating protein to terminate GPCR signaling by dephosphorylating the G_α_•GTP subunit [78,79]. Silencing RGS1 restored chemotaxis of cytotoxic T cells and Th1 cells to enhance their infiltrating toward tumors. This process revealed that a G protein component, G_α_, is essential for the chemotaxis of cytotoxic T cells and Th1 cells [80]. The regulatory proteins of GPCR would be potential drug targets to revive anti-tumor activity.

## 3. The Expression and Implications of CXCL13/CXCR5

CXCL13 is a chemoattractant that selectively interacts with its receptor, CXCR5, to promote the migration of CXCR5^+^ cells toward high CXCL13 concentration areas. CXCL13 was originally identified as B cell attracting chemokine 1(BCA-1) and was shown to act on CXCR5^+^ B cells to induce chemotaxis and Ca^2+^ mobilization [81]. In lymph nodes, the CXCL13/CXCR5 axis is essential for homing B lymphocytes to lymphoid tissue, and CXCL13/CXCR5 deficient mice show the disrupted localization of B cells [81].

CXCL13 recruits B cells to tumors, where they form tertiary lymphoid structures (TLSs) [82]. TLSs are similar to secondary lymphoid organs on the cellular, morphological, and molecular levels, and TLSs are considered with good prognostic markers, commonly associated with better survival rates. However, the mechanisms underlying TLS development remain unclear. Rodriguez et al. found that the organization of cancer-associated fibroblasts into reticular networks depends on tumor necrosis factor receptor signaling, which acts as a lymphoid tissue organizer [83]. Cancer-associated fibroblasts secrete CXCL13 to recruit CXCR5^+^ B cells expressing lymphotoxin-α1β2, which expands TLSs in the tumor microenvironment. Moreover, CD8^+^ T cells mediate cancer-associated fibroblast organization, serving as an inducer of lymphoid structures to drive the formation of TLSs.

CXCR5^+^ T cells were originally defined as CD4^+^ memory-like T cells, which function to assist B cells with antibody production. The colocalization of CXCL13, CXCR5^+^ T cells, and B cells was detected in the follicular mantle zone. A unique subset of T cells that assist in B cell function was identified as T_FH_ cells [84]. High levels of BCL6 drive T_FH_ differentiation, and Blimp-1 is able to block T cells from differentiating into T_FH_ cells [71].

T_FH_ cells are the major CXCL13-producing cells in the lymphoid structure. In tumor microenvironments, tumor-infiltrating CXCL13-producing T_FH_ cells may be involved in promoting local B cell localization, differentiation, and maturation [85]. An activated germinal center of B cells then contributes to the formation of TLSs in tumors. Gu-Trantien et al. first observed CXCL13 producing T_FH_ cells in breast cancer and found that the T_FH_ signature strongly predicted a positive clinical outcome in breast cancer [86]. Naïve CD4^+^ T cells differentiate into T helper 1 (Th1)-oriented T_FH_ cells that produce CXCL13 in tumor microenvironments after being primed by antigen-presenting cells in the lymph node [87,88]. The balance of T_FH_/Th1 differentiation from precursor cells is determined by the competitive expression of Bcl6 and T-bet [71,89,90,91]. Further investigation revealed that tumor-infiltrating T_FH_ cells produce CXCL13 to attract CXCR5^+^ CD8^+^ T cells and CXCR5^+^ B cells toward the germinal centers within the TLS, where both T cells and B cells are primed, reinforcing their cytotoxic capacity against cancer cells [92]. These studies indicate that BCL6-dominant signaling promotes CXCL13 expression in T_FH_ cells [93]. Another subset of T_FH_ cells, which were PD-1^hi^CXCR5^−^CD4^+^ T cells that expressed CXCL13, did not show elevated BCL6 expression.

In addition to T_FH_ cells, there are several CXCL13 producing cells in lymphoid tissue and would secrete CXCL13 to chemoattract cells that express CXCR5. Denton et al. identified pulmonary fibroblasts as CXCL13-producing cells induced by Type I interferon (Type I IFN) during Influenza A virus transfection, which facilitate the recruitment of CXCR5^+^ B cells and T cells to the lungs to generate pulmonary germinal centers [94].

Antigen-specific CD4^+^ T cells mediate and coordinate immune cell functions in antitumor activities. Although the effects of ICI have been thoroughly explored in CD8^+^ T cells, the roles played by CD4^+^ T cells in response to ICI remain poorly understood. Balança et al. studied exhausted CD4^+^ T cells in head and neck, cervical, and ovarian cancers [95]. With exhausted CD4^+^ T cells defined as those cells presenting high PD-1 and CD39 levels, which were found to secrete CXCL13 and express the transcription factor thymocyte selection-associated high mobility group box (TOX). The transcription factor SRY-box transcription factor 4 (SOX-4) mediates CXCL13 production on CD4^+^ T cells, enriching transforming growth factor-β (TGF-β) and reducing IL-2 expression [96]. Similar to CXCL13^+^ exhausted CD8^+^ T cells, exhausted CD4^+^ T cells recruit B cells and promote the formation of TLS, suggesting a good response to immunotherapy and indicating a crucial role for CD4^+^ T cells in mediating the functions of various immune cells.

Cytotoxic CD8^+^ T cells are the strongest effectors, playing important roles in the anticancer immune response. When examining the CXCL13/CXCR5 axis, Workel et al. found that TGF-β dependent CD103^+^CD8^+^ tumor-infiltrating T cells represent an important CXCL13 resource [97]. When TGF-β receptors are inhibited, CXCL13 signaling is abrogated. CXCL13^+^CD103^+^CD8^+^ tumor-infiltrating T cells are associated with B cell recruitment, TLS formation, and neoantigen burden. Additionally, Thommen et al. also found that PD-1^high^CD8^+^ tumor-infiltrating T cells produce more CXCL13 than PD-1^-^CD8^+^ T cells in non-small-cell lung cancer (NSCLC) [34]. They also observed that CXCL13 recruited other CXCR5-expressing immune cells and induced the formation of TLSs in NSCLC. PD-1^high^CD8^+^ tumor-infiltrating T cells are enriched inside the TLS, and their presence successfully predicts the response to anti-PD-1 and is positively correlated with overall survival.

In chronic hepatitis B infection, Li et al. found that higher levels of CXCL13 facilitate the recruitment of CXCR5^+^CD8^+^ T cells to liver tissue in patients, which is associated with a favorable response to antiviral drug treatment [98]. CXCR5^+^CD8^+^ T cells secrete hepatitis B virus (HBV)-specific cytokines, such as IL-2, IFN-γ, IL-17, and IL-21. The subset of CD8^+^ T cells is partially exhausted but not dysfunctional and is involved in controlling the viral infection load. Strikingly, the adoptive transfer of CXCR5^+^CD8^+^ T cells to an HBV mouse model resulted in a significant decrease in HBV antigen expression. Finally, they found that B cell-deficient mice have a lower frequency of CXCR5^+^CD8^+^ T cells and decreased the HBV-specific IFN-γ^+^ CXCR5^+^CD8^+^ T cells in the blood and liver. These data demonstrated that B cells are required for CXCR5^+^ CD8^+^ T cells, contributing to the functions and activities of CXCR5^+^CD8^+^ T cells.

In classical Hodgkin lymphoma, Le et al. identified a special CD8^+^ T cell subset expressing CXCR5 and an inducible T cell costimulator (ICOS) [99]. This subset was functionally similar to T_FH_ cells, with low CCR7 expression and high levels of BCL6, PD-1, CD200, and OX40 expression. In addition, these cells displayed poor cytotoxic function and low interferon-secretion and produced IL-4, IL-21, and CXCL13, similar to CD4^+^ T_FH_ cells. Gene profiling analyses demonstrated that CXCR5^+^ICOS^+^CD8^+^ T cells are significantly similar to CD4^+^ T_FH_ cells and are involved in the generation of lymphoma tumors with residual germinal centers. Overall, CD8^+^ T cell differentiation pathways are functionally similar to those involved in CD4^+^ T_FH_ cell differentiation in Hodgkin lymphoma.

Follicular dendritic cells represent the primary cellular source of cytokines in lymphoid organs [100]. Previous research has identified that various dendritic cell subsets are able to secrete CXCL13, playing an important role in the establishment of interactions between lymphocytes and dendritic cells [101]. Additionally, CXCL13 serves as a plasma biomarker that reflects germinal center activity [102]. Follicular dendritic cells represent the primary source of CXCL13 expression in reactive tonsils and lymph nodes located in the B cell zone. Moreover, dysplastic and neoplastic follicular dendritic cells are able to secrete CXCL13 to recruit lymphocytes to areas of dense inflammatory infiltration [100]. Overall, dendritic cells secrete CXCL13 to recruit CXCR5^+^ lymphocytes, orchestrating the immune response in lymphoid tissues.

**Figure 2:**
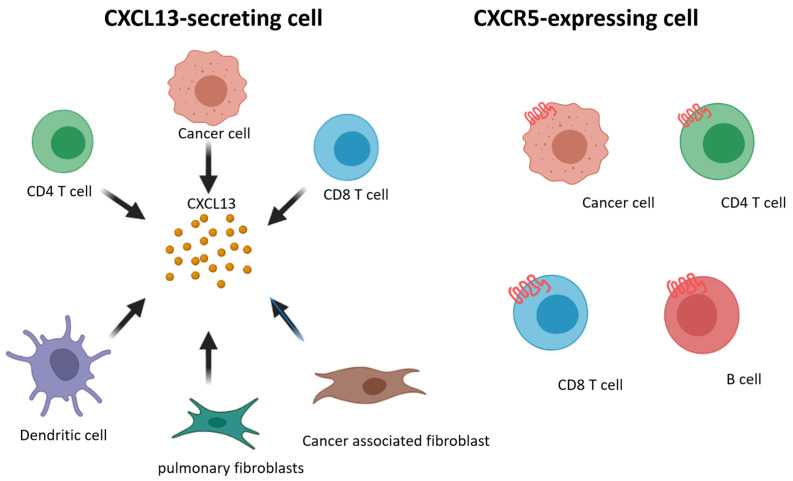
Schematic representation of CXCL13 and CXCR5 expressing on different cell subsets.

**Figure 3:**
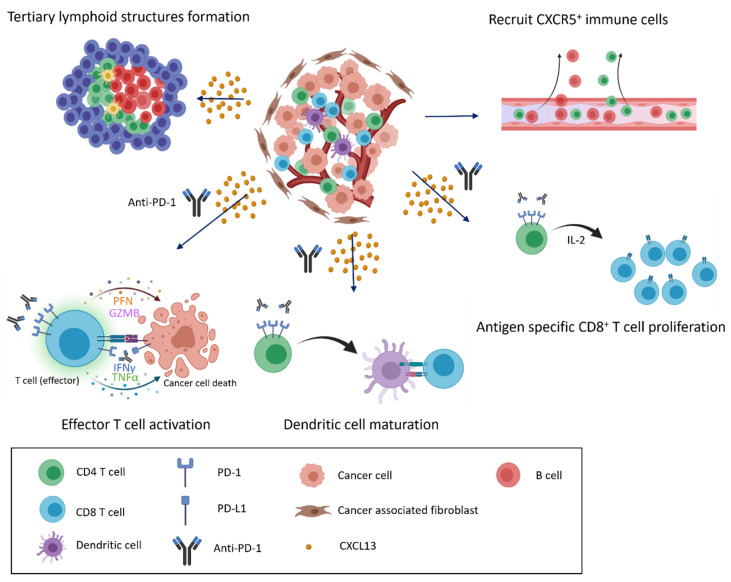
CXCL13/CXCR5 signaling and response to immune checkpoint blockade in the tumor microenvironment. CXCL13 secretion and enrichment in the tumor microenvironment alter the immune cell composition. CXCL13 recruits cells that express CXCR5 to infiltrate the cancer microenvironment, inducing the formation of tertiary lymphoid structures and the further infiltration of various immune cells (**top**). When anti-PD-1 antibodies are present, recruited immune cells actively attack cancer cells, and exhausted cells transition into effector cells, leading to the proliferation of CD8^+^ T cells and the maturation of dendritic cells in response to anti-PD-1 treatment (**bottom**).

We summarize prior findings regarding CXCL13 and CXCR5 expression. CD8^+^ and CD4^+^ T cells, cancer-associated fibroblasts, cancer cells, and dendritic cells are able to secrete CXCL13, and CD8^+^ and CD4^+^ T cells, B cells, and cancer cells express CXCR5 (Figure 2). Increasing research has reported the detection of CXCL13 in the tumor microenvironment of many different types of cancer [103,104,105,106,107,108,109]. When the cancer microenvironment is enriched in CXCL13, the recruitment of CXCR5-expressing leukocytes to the tumor microenvironment increases. B cells are recruited by CXCL13 to promote TLS formation in the tumor microenvironment (Figure 3). Previous studies have correlated TLS formation with better prognosis among patients with cancer [110,111,112,113]; therefore, treatments that promote the development of TLSs represent potential therapeutic strategies.

## 4. The CXCL13/CXCR5 Signaling Axis in the ICI Response of Preclinical Models

Immune checkpoint blockade therapy has been hugely successful with effective results in many cancer types [114,115,116], acting through reinforcement of T cell function and improving the cytotoxic capacity against cancer cells [1]. In this section, we summarize existing research examining the contributions of CXCL13/CXCR5 to immunotherapy in preclinical models (Table 1).

Li et al. used an HBV mouse model and HBV-related hepatocellular carcinoma (HCC) samples from patients to investigate the role placed by CXCL13/CXCR5 signaling in HBV and HBV-related HCC [98]. They detect CXCR5 and CXCL13 expression levels using RNA-sequencing, quantitative reverse transcriptase PCR (qPCR), immunohistochemistry (IHC), enzyme-linked immunosorbent assay (ELISA), and Western blotting methods. They identified the recruitment of CXCR5^+^CD8^+^ T cells by CXCL13. Additionally, anti-PD-1 blockade and recombinant IL-21 therapy can promote the secretion of IFN-γ from CXCR5^+^CD8^+^ T cells in patients with HBV. In an HBV mouse model, CXCR5^+^CD8^+^ T cells would inhibit hepatitis B surface antigen (HBsAg) expression, which was not observed in response to CXCR5^-^CD8^+^ T cells. Anti-PD-1 and IL-21 treatment strengthen the function of immune cells involved in HBV clearance or the attack of cancer cells.

Rodriguez et al. studied cancer-associated fibroblasts to determine the effects of TLSs in a melanoma and colon adenocarcinoma mouse model [83]. Anti-PD-L1 and a combination of anti-PD-1 and anti-CTLA4 both increase the formation of TLSs in the tumor microenvironment, resulting in reduced tumor growth, and increased T cell recruitment to the tumor, as indicated by the formation of discrete T cell and B cell zones. They observed CXCL13 expression on cancer-associated fibroblasts, as assessed by immunofluorescence and real-time PCR. Finally, they found that immunotherapy increased the tumor-associated TLS size and number, which was associated with reduced cancer growth.

Balança et al. studied the role of CD4^+^ T cells in immunotherapy during the attack on cancer cells [95]. They applied single-cell RNA sequencing analysis to patient-derived samples of head and neck, cervical, and ovarian cancer to detect the expression of transcription factor TOX and the chemokine CXCL13. They found that anti-PD-1 therapy promoted the functional activity of exhausted CD4^+^ T cells, promoting dendritic cell maturation and CD8^+^ T cell proliferation. Exhausted CD4^+^ T cells also play important roles in regulating and coordinating immune cells in the attack on cancer cells after immunotherapy treatment.

Yang et al. analyzed the influence of CXCL13 on the ICI response in an ovarian cancer mouse model [117]. They identified different immune cell subsets in samples from patients with high-grade ovarian cancer that were able to secrete CXCL13. High levels of CXCL13 expression resulted in increased lymphocytes infiltration in the tumor, as assessed by immunofluorescence. Conclusively, anti-PD-1 combined with CXCL13 treatment is associated with a better treatment response than anti-PD-1 alone in a mouse model of ovarian cancer.

Zhang et al. combined the inhibitor of cyclin-dependent kinases 4 and 6 (CDK4/6i) with anti-PD-1 therapy and examined the synergistic effects in an ovarian cancer mouse model [119]. PCR and cytokine array methods were applied to detect cytokine and chemokine levels, which revealed that ID8 ovarian cancer cells secrete higher levels of CXCL10 and CXCL13 after CDK4/6i treatment resulting in increased lymphocytes infiltration in the tumor microenvironment. Combination treatment is associated with a better response and more active function among CD8^+^ and CD4^+^ T cells than anti-PD-1 or CDK4/6i alone, and the synergistic antitumor effects were dependent on the activities of B cells and CD8^+^ T cells.

B cells play important roles in the results of immunotherapy. Cabrita et al. reported that TLSs influence the immunotherapy response of melanoma, showing that the co-occurrence of CD8^+^ T cells and CD20^+^ B cells can predict the response to ICI therapy in patients with metastatic melanomas [118]. CXCL13, CXCR5, CD8^+^ T cells, and CD20^+^ B cells colocalized in melanoma samples obtained from patients using immunofluorescence staining, which revealed the TLS formation in these tumors. Moreover, gene expression associated with TLS formation was able to predict the clinical outcomes of immunotherapy. Without TLS formation, a dysfunctional molecular phenotype can be observed, with adverse effects on immunotherapy outcomes. TLSs play critical roles in influencing the response of the tumor microenvironment to ICI therapy, and therapeutic strategies that induce TLSs’ formation might improve the efficacy of immunotherapy.

CXCL13 has been identified as an exhaustion marker in previous research [120,121]. Studies have increasingly identified immunotherapy as being positively correlated with exhausted immune cells [122,123,124], including our research results for HCC [125]. The increased expression of exhaustion markers in the tumor microenvironment is an indicator of increased immune cell infiltration, which suggests a good response to immunotherapy. Bassez et al. identified several genes that were able to predict a better immune response to anti-PD-1 treatment associated with CXCL13 [126]. CXCL13 expression at high levels corresponds with T cells expansion after anti-PD-1 treatment for breast cancer, which is associated with a good response to immunotherapy.

Research continues into understanding why some subsets of patients respond to immunotherapy, but others do not respond. The heterogenicity of tumor cells and immune cells is a very important issue, which brings about different immunotherapy efficacy regardless of tumor types. The study divided cancer patients into two groups [126]. One group had expanding T cells, and the other had non-expanding T cells following anti-PD-1 therapy. The results demonstrated that CXCL13 expression on T cells is associated with the expansion of T cells, which implies that one subset of patients has a good response after immune checkpoint inhibitors treatment. The research sheds light on the exploration of the heterogenicity of immunotypes as well as associated genes for immunotherapy response.

We summarize the effects of CXCL13 and CXCR5 expression on preclinical models of the immunotherapy response. TLSs play critical roles in influencing the response to immune checkpoint blockade in the tumor microenvironment. Anti-PD-1 antibodies in the tumor microenvironment activate CD8^+^ T cells, and CD4^+^ T cells mediate CD8^+^ T cell proliferation and dendritic cell maturation (Figure 3). CXCL13 expression recruits additional immune cells to infiltrate the tumor environment to attack cancer cells following immunotherapy treatment.

## 5. CXCL13/CXCR5 Axis for ICI Response in Clinical Tumors

Immune checkpoint blockade can regulate tumor progression. Compared with chemotherapy, ICI therapy has a superior duration of response (20.4 months vs. 6.3 months) [127,128]. However, only 15–20% of patients benefit from immunotherapy [128] and the identification of biomarkers able to predict therapeutic response to ICI. Recently, some biomarkers were identified that were able to accurately predict the response of cancer patients to PD-1 blockade. Le et al. proposed that patients with mismatch repair deficiencies have a higher response rate to pembrolizumab treatment than patients with proficient mismatch repair processes [129]. In this section, we summarize clinical trials and research linking the CXCL13/CXCR5 axis with the ICI response in patients (Table 2).

### 5.1. Breast Cancer

Gu-Trantien et al. analyzed CD4^+^ T cells in tumor tissues by profiling gene signatures. They identified T_FH-_related genes, such as CD200, CXCL13, ICOS, and PD-1, that were over-expressed in infiltrated tumors. They divided patients’ tumors into those with a high level of CD4^+^ tumor-infiltrating lymphocytes (TILs) and those with minimal infiltration [86]. Among patients with a high level of TILs, CXCL13 was the most highly overexpressed gene. IHC staining indicated that CXCL13 was not expressed in tumor cells but was detected intensively in TLSs and CD4^+^ T_FH_ cells. Clinical correlations with CXCL13 were performed by investigating the disease-free survival (DFS) of 794 breast cancer patients, which revealed that CXCL13 expression was positively correlated with DFS in breast cancer, especially in the human epidermal growth factor 2 (HER2^+^) group. The authors also analyzed a cohort of 996 patients with breast cancer who received neoadjuvant chemotherapy, which showed that CXCL13 expression was highly correlated with a complete response in HER2^+^ patients.

Zhang et al. designed a clinical trial to identify key immune subpopulations associated with clinical outcomes for anti-PD-L1 blockade therapy in triple-negative breast cancer (TNBC) [131]. Twenty-two patients with late-stage TNBC were enrolled in this cohort, divided into two groups with equal numbers. Patients in one group were treated with paclitaxel, whereas the other group received paclitaxel plus atezolizumab. After 4 weeks, tumor biopsies and peripheral blood cells were collected from both groups. TILs were collected and analyzed by single-cell RNA-sequencing, single-cell ATAC-sequencing, and TCR-sequencing. In this cohort, the authors developed two indexes to associate immune subsets with clinical benefits in TNBC: the predictive index (Pi) and the therapeutic index (Ti). The Pi correlated the compositions of TIL subsets with changes in tumor volume, whereas the Ti correlated between TIL subsets dynamics with tumor volume changes. They identified B cells as being the most predictable subset for predicting the response to immune checkpoint blockade in the Pi analysis, whereas T cells were the most predictable subset to predict immune checkpoint blockade in the Ti analysis, indicating a functional change in T cells during ICI therapy. The authors clustered T cells using a high-resolution T cell map, which identified expanded CXCL13^+^CD8^+^ and CXCL13^+^CD4^+^ populations in patients who responded to paclitaxel plus atezolizumab but not patients who did not respond to treatment. Furthermore, the analysis of CXCL13^+^CD8^+^ T cells using a single-cell assay for transposase-accessible chromatin using sequencing (ATAC-seq) showed that CXCL13^+^CD8^+^ cells had more accessible chromatin regions in the *IFNG*, *GZMK*, and *PDCD1* loci.

### 5.2. Bladder Cancer

Goswami et al. analyzed the combined data from two clinical trials, CheckMate275 and IMvigor210, to determine the correlations of the AT-rich interactive domain containing protein 1A (ARID1A) mutations and CXCL13 expression with the patient’s response to immune checkpoint blockade therapy [109]. CheckMate 275 is a phase 2 trial of nivolumab for the treatment of metastatic urothelial carcinoma (*n* = 265). IMvigor210 is a phase 2 trial of atezolizumab for the treatment of advanced or metastatic urothelial bladder cancer. They identified that an ARID1A mutation alone or CXCL13-high expression is correlated with favorable responses to ICI, and ARID1A mutation in combination with high levels of CXCL13 expression could predict the better response of patients to immune checkpoint blockade therapy than ARID1A mutation in combination with low levels of CXCL13 or high levels of CXCL13 in combination with ARID1A wild-type.

### 5.3. Non-Small Cell Lung Cancer

Thommen et al. classified CD8^+^ TILs derived from patients with NSCLC into three groups based on their PD-1 surface-level expression [34]. PD-1^-^ were CD8^+^ TILs without detectable PD-1 expression; PD-1^N^ were CD8^+^ TILs with PD-1 levels similar to those observed in healthy donors; PD-1^T^ were CD8^+^ TILs PD-1 with considerably high expression levels. The reactive capacity of PD-1 TILs was confirmed by co-culturing PD-1^T^, PD-1^N^, and PD-1^-^, respectively, with autologous tumors isolated from eight patients. Among the eight patient-derived tumors, PD-1^T^ TILs showed strong reactivity against the tumor cells in 6 of 8 cultures. Gene expression diversity among PD-1^T^, PD-1^N^, and PD-1^-^ populations was identified by transcriptome analysis, which revealed that CXCL13 was one of the most upregulated genes in the PD-1^T^ subset. The authors used bead-based immunoassays to quantify inflammatory cytokine and chemokine levels in sorted PD-1^T^ TILs after 24h of culture, identifying that the levels of CXCL13 were the highest in the array. These data indicated that PD-1^T^ TILs are likely to secrete CXCL13 into the tumor microenvironment. Of 21 stage IV NSCLC patients receiving anti-PD-1 therapy, 7 patients were identified as responders, whereas the other 14 patients were identified as non-responders. Responders were characterized by TIL subsets with a higher percentage of PD-1^T^ TILs and total PD-1^T^ TIL cell numbers. Those with PD-1^T^ subsets > 1% of total cells were associated with longer median survival than those with PD-1^T^ subsets < 1% of total cells following the administration of anti-PD-1 therapy. IHC and digital image analysis suggested that PD-1^T^ TILs were localized in TLSs that formed both intratumorally and peritumorally. Intensive B cell marker staining and CD4^+^ T_FH_ markers colocalized with PD-1^T^ TILs, implying that CD8^+^PD-1^T^ TILs might recruit CXCR5^+^ B cells and T_FH_ cells into the tumor region and that B cells, as well as T_FH_ cells, could reinforce the antitumor capacity of CD8^+^ T cells.

### 5.4. Hepatocellular Carcinoma

Liver cancer is one of the world’s most common cancers and the second leading cause of cancer deaths, and we also have some related studies in the past [125,134,135]. Hsu et al. established a platform to assess various biomarkers associated with prognostic efficacy for the immunotherapy response of patients with hepatocellular carcinoma (HCC) using Nanostring RNA analysis and multiplex immunofluorescence staining methods [125]. In this cohort, RNA was isolated from tumor samples before patients received anti-PD-1 or combination anti-PD-1 and anti-PD-L1 blockade therapy (*n* = 42). RNA was then hybridized to nCounter^®^ probes for the 770 predefined genes featured in the PanCancer Immune Profiling Panel. They found that exhausted CD8^+^ T cells were enriched in responders and analyzed exhausted CD8^+^ T cells using transcriptome analysis. In this Immune Profiling Panel, nine genes expressed on exhausted T cells were positively correlated with a better response to anti-PD-1 monotherapy or combination anti-PD-1 and anti-PD-L1 therapy, including CXCL13 gene expression. In another study, the exploratory analysis identified distinct gene signatures associated with tumor response and resistance to anti-PD-1 monotherapy in HCC patients also showed that CXCL13 was positively correlated with a better response to anti-PD-1 therapy [136].

### 5.5. Pan-Cancers

Litchfield et al. collected exome/transcriptome data from 12 cohorts, including 1008 patients who received immune checkpoint blockade therapy for the treatment of urothelial cancer (*n* = 387), melanoma (*n* = 353), head and neck cancer (*n* = 107), non-small cell lung cancer (*n* = 76), renal cell carcinoma (*n* = 51), colorectal cancer (*n* = 20), and breast cancer (*n* = 14) [132]. Immune checkpoint blockade could be classified into (1) anti-PD-1 (*n* = 432), (2) anti-PD-L1 (*n* = 421), and anti-CTLA4 (*n* = 155). The authors collected raw fastq sequencing data from these 12 cohorts and reprocessed them using a uniform bioinformatics pipeline. Next, they obtained clinical response data for participants in each of the 12 cohorts and reclassified them into complete response (CR)/partial response (PR) group versus stable disease (SD)/progressive disease (PD). Using the CPI1000+ database v1.1, they identified 101 genes that were significantly upregulated in CR/PR versus SD/PD, among which CXCL13 was the most upregulated gene among responders (CR/PR), suggesting that CXCL13 expression may be correlated with a patient’s response to ICI therapy.

The incidence of ICI-induced mortality is estimated at about 0.3% to 1.3% [137], and the risk is lower than conventional therapy, such as 0–4% with targeted therapies [138] and about 15% with allogeneic hematopoietic stem cell transplantation [139]. Different doses [140,141] and the timing of administration and agents [142] may promote different levels of immune-related adverse events (irAEs) on patients receiving ICI. Common irAEs include headache, rash, pruritus, fatigue, pneumonitis, diarrhea, arthralgia, and endocrinopathies, which usually involve multiple organs [143,144,145]. Future research must address irAEs through more clinical trials, medical findings, and cutting-edge diagnosis tools, and the detailed mechanism causing these events will be better understood.

Two-thirds of CD8^+^ TILs express PD-1, and they mainly present an exhaustion situation [146]. ICI therapy is able to restore and enhance T cell function and effector cytokine production, such as TNF-α, IL-2, and IFN-γ [146,147,148]. Additionally, PD-1 inhibition could reverse the exhaustion status of specific T cells in tumor microenvironments [148].

We summarize the previous findings examining the association between CXCL13/CXCR5 expression and the clinical response to tumor immunotherapy. CXCL13 expression was able to predict the response to ICI by directly or indirectly influencing the responses of various cancer types. However, the mechanism through which CXCL13/CXCR5 signaling induces the response of various immune subsets to ICI treatment remains unknown, and determining the cellular subsets that serve as the major producers of the CXCL13/CXCR5 signal that induces immune cell infiltration in cancer requires additional study.

## 6. Conclusions and Perspectives

The CXCL13/CXCR5 signaling axis is able to recruit immune cells and enhance the capacity to attack cancers. On the other hand, the CXCL13/CXCR5 axis may induce tumor development and facilitate the downregulation of T cell immunity due to associated immunosuppression cells infiltrating within the tumor microenvironment, such as MDSC [149] and T_reg_ [150]. CXCL13/CXCR5 signaling forms two opposite influences for cancer development. When two distinct pathways have similar strengths, cancers maintain homeostasis. However, anti-PD-1 is able to enhance the function of T cells, strengthening their anti-tumor ability. Additionally, MDSC and T_reg_ express PD-L1 [151], and they may suppress T cell response by the PD-1/PD-L1 pathway. ICI therapy reduces immunosuppression and increases immune response, which is able to control tumor growth.

CXCL13 is a very important factor involved in the recruitment of CXCR5-producing immune cells to infiltrate the tumor microenvironment. CXCL13 expression was able to induce the establishment of TLS, which presents tumor antigens to T cells to determine consecutive T and B cell responses, resulting in the generation of effector T cells, plasma cells, and antibodies. In other words, the CXCL13/CXCR5 signal is able to turn “cold tumors” into “hot tumors”. The ability of immunotherapy indicates an improved response, which activates lymphocyte function to kill cancer cells. The validation of the CXCL13/CXCR5 signaling pathway components as useful biomarkers for cancer diagnosis or prognosis, particularly the response to ICI, requires further investigation. Exploring the regulation of this pathway could provide an avenue for the design or more effective immunotherapy-based treatment strategies for cancer.

## Figures and Tables

**Table 1:** Cancer immunotherapy approaches related to the CXCL13/CXCR5 axis, based on preclinical models.

Target in the Axis	Treatment	Disease	Experimental Method	Method of Detection	Value	Outcome
CXCR5^+^ CD8^+^ T cells	IL-21 Anti-PD-1	HBV-related HCC	Ex vivo from patients; in vivo in mice	RNA-seq qPCR IHC ELISA Western	Favorable	CXCR5^+^CD8^+^ T cells are recruited to the liver, aiding antibody production and controlling the viral load. Anti-PD-1 and IL-21 treatment restore CXCR5^+^CD8^+^ T cell function [98].
PD-1^hi^ CXCL13^+^ CD39^+^CD4^+^ T cells	Anti-PD-1	Head and neck cancer, cervical cancer, and ovarian cancer	Ex vivo from patients	scRNA-Seq	Favorable	PD-1 blockade evokes CD39^+^CD4^+^ T cell function and improves dendritic cell maturation and CD8^+^ T cell proliferation [95].
CXCL13^+^ immune cells	Anti-PD-1 CXCL13	Ovarian cancer	In vivo in mice (subcutaneous)	Immunofluorescence IHC ELISA	Favorable	CXCL13 increases CD8^+^ T cell infiltration at the tumor site and upregulates effector cytokine levels. CXCL13 enhances the anti-PD-1 response [117].
CXCR5^+^ CXCL13^+^ B cells	Anti-PD-1 Anti-CTLA4	Metastatic melanoma	Patients’ tumor samples	IHC Immunofluorescence	Favorable	The co-occurrence of CD20^+^ B cells and CD8^+^ T cells is associated with better survival. Tertiary lymphoid structure formation containing CD8^+^ T cells and CD20^+^ B cells predicts clinical outcomes for immune checkpoint inhibitors [118].
ID8 cells (cancer cells) secreting CXCL13	Combination of CDK4/6i and anti-PD-1	Ovarian cancer	In vivo in mice (ip)	RT Profiler PCR array	Favorable	CDK4/6 inhibition (abemaciclib) enhances CD8^+^ T cell, and B cell infiltration in a murine ovarian cancer model induces pro-inflammatory responses and increases CXCL13 secretion, which recruits additional lymphocytes to the tumor microenvironment. CDK4/6 inhibition and anti-PD-1 combination improve treatment efficacy in ovarian cancer [119].
Cancer-associated fibroblasts expressing CXCL13	Anti-PD-L1 Anti-CTLA4	Melanoma and colon adenocarcinoma	In vivo in mice (ip, subcutaneous)	Real-time PCR Immunofluorescence	Favorable	Cancer-associated fibroblasts depend on tumor necrosis factor receptor signaling to orchestrate tumor-associated TLS development, and CD8^+^ T cells organize cancer-associated fibroblasts into reticular networks. The number and size of tumor-associated TLSs with discrete B and T cells are associated with favorable responses to immune checkpoint blockade [83].

CXCL, CXC chemokine ligand; PD-1, programmed cell death protein 1; CTLA4, cytotoxic T lymphocyte-associated protein 4; HBV, hepatitis B virus; HCC, hepatocellular carcinoma; ip, intraperitoneal; RNA-seq, RNA-sequencing; qPCR, quantitative real-time reverse transcriptase-polymerase chain reaction; scRNA-seq, single-cell RNA-sequencing; IHC, immunohistochemistry; ELISA, enzyme-linked immunosorbent assay; TLS, tertiary lymphoid structure.

**Table 2:** Cancer immunotherapy approaches relative to the CXCL13/CXCR5 axis based on clinical data.

Target in the Axis	Treatment	Disease	Method of Detection	Number of Patients Investigated	Value	Outcome
CXCL13^+^PD1^+^CD8^+^ T cells	Anti-PD-1	Non-small cell lung cancer	Transcriptome analysis	Peripheral blood of healthy donors (*n* = 6) Fraction of PD-1^bright^ within CD8^+^ TILs (*n* = 24)	Favorable	The presence of PD-1^+^CD8^+^ T cells can predict PD-1 blockade response and survival rate [34].
CXCL13	Anti-PD-1 Anti-PD-L1	Metastatic urothelial carcinoma and bladder cancer	Whole-exome sequencing data analysis TCGA analysis	CheckMate275 (*n* = 270) IMvigor210 (*n* = 310)	Favorable	CXCL13 expression plus ARID1A mutation work together to predict a favorable response to anti-PD-1 blockade [109].
CXCL13	Anti-PD-L1	Bladder cancer	Single-sample GSEA Gene ontology analysis KEGG analysis WGCNA	IMvigor210 (*n* = 310)	Favorable	CXCL13 expression plus TLS formation predict a favorable response to anti-PD-1 blockade [130].
CXCL13^+^/LAG3^+^CD8^+^ T cells	Anti-PD-1 Anti-PD-L1	Hepatocellular carcinoma	Multiplex immunofluorescence staining TCGA-LIHC analysis Nanostring RNA analysis	Cohort 1 (*n* = 24) Cohort 2 (*n* = 18)	Favorable	CXCL13 expression plus exhausted T cells marker expression predict a favorable response to anti-PD-1 blockade [125].
CXCL13^+^CD8^+^ T cells CXCL13^+^CD4^+^ T cells	Anti-PD-1 Nab-Paclitaxel	Triple-negative breast cancer	ATAC-seq RNA-seq Single-cell RNA seq Whole-exome sequencing IHC	*n* = 22	Favorable	High levels of baseline CXCL13^+^ T cells predict favorable response to anti-PD-L1 plus nab-paclitaxel combination therapy [131].
CXCL13 in CD8^+^ T cells	Anti-PD-L1, Anti-PD-1, Anti-CTLA4	Seven cancer types	Single-cell RNA-seq ATAC-seq	*n* = 1008	Favorable	CXCL13 expression is a marker of clonal neoantigen-specific CD8^+^ TILs that selectively expresses in CPI responders (“CR/PR”). [132].
CXCL13 in tumor cells	Anti-PD-1	Pan-cancer	Nanostring RNA analysis IHC Gene expression profiles	NCT01295827 (*n* = 1260) NCT01848834 (*n* =297) NCT02054806 (*n* = 477)	Favorable	T cells expanded signature including CXCL13 and 17 other genes are necessary for clinical response to PD-1 checkpoint blockade [133].

CXCL, CXC chemokine ligand; PD-1, programmed cell death protein 1; PD-L1, programmed death-ligand 1; CTLA4, cytotoxic T lymphocyte-associated protein 4; RNA-seq, RNA-sequencing; ATAC-seq, Assay for Transposase-Accessible Chromatin with high-throughput sequencing; CPI, checkpoint inhibitor; HCC, hepatocellular carcinoma; TLS, tertiary lymphoid structure; ARID1A, AT-rich interactive domain-containing protein 1A; TIL, tumor-infiltrating lymphocyte; IHC, immunohistochemistry; WGCNA, Weighted correlation network analysis; TCGA, The Cancer Genome Atlas; TCGA-LIHC, The Cancer Genome Atlas Liver Hepatocellular Carcinoma; KEGG, Kyoto Encyclopedia of Genes and Genomes.
